# Treatment of Plantar Warts with a Nitric-Zinc Complex Solution: A Review of 72 Cases

**DOI:** 10.3390/v16081307

**Published:** 2024-08-16

**Authors:** Sara Garcia-Oreja, Francisco Javier Alvaro-Afonso, Paula Vigara-Aranda, Guillermo Paramio-Rodriguez, Diego Leon-Herce, Jose Luis Lazaro-Martinez

**Affiliations:** 1 Facultad de Enfermería, Fisioterapia y Podología, University Podiatric Clinic, Complutense University of Madrid, 28040 Madrid, Spain; sagarc14@ucm.es (S.G.-O.); paulavig@ucm.es (P.V.-A.); guiparam@ucm.es (G.P.-R.); dileon01@ucm.es (D.L.-H.); diabetes@ucm.es (J.L.L.-M.); 2 Instituto de Investigación Sanitaria del Hospital Clínico San Carlos (IdISSC), University Podiatric Clinic, Complutense University of Madrid, 28040 Madrid, Spain

**Keywords:** cutaneous warts, human papillomavirus, nitric–zinc complex solution, plantar warts

## Abstract

Background: There are multiple conservative treatment options for plantar warts, but none have proven to be universally effective. Nitric acid is often used empirically by podiatrists in the treatment of plantar warts. A novel medical device or topical solution of nitric–zinc complex solution (NZCS) could potentially offer an effective and safe alternative for the targeted treatment of plantar warts. Objective: To observe the rate of complete healing of NZCS in a series of plantar wart cases and to establish the minimum number of product applications and time needed for healing. This will help standardize and protocolize its use. Methods: A descriptive study was conducted involving 72 patients who exhibited symptoms of plantar warts. These patients underwent chemical treatment using a nitric–zinc complex. Results: The cure rate with NZCS was 59.2%. The average number of NZCS applications was 5.9 ± 3.0 and the mean duration of treatment was 9.4 ± 7.1 weeks. A recurrence rate of 6.7% was observed. Conclusions: The topical solution of the nitric–zinc complex is an effective treatment for plantar warts, which can be considered a first-line treatment option in the general population.

## 1. Introduction

Plantar warts are benign skin lesions caused by the Human Papilloma Virus (HPV) [1]. Over 350 subtypes of this virus have been described [2], with subtypes 1, 2, 4, 10, 27, and 57 being the most prevalent in the foot [3,4]. The overall prevalence of HPV is estimated at 40%, with the annual incidence of plantar warts at approximately 14% [5].

There are various conservative treatment options for plantar warts, including cryotherapy, salicylic acid in different concentrations (10–70%), cantharidin 1%, bleomycin intralesional 0.1%, immunotherapy, and laser [6]. However, none have proven to be consistently effective in all patients [6,7]. Topical treatments employing physical and chemical products aim to destroy the affected epidermal cells [1]. However, they sometimes fail to entirely eliminate the latent virus in adjacent cells. This results in the persistence and recurrence of warts, thereby complicating their treatment [8,9].

Hekmatjah et al., in a recent systematic review, concluded that despite their lower efficacy compared to newer treatments such as laser therapy [10], cryotherapy and salicylic acid remain the primary treatments for plantar warts. Another recent systematic review revealed that the cure rates for cryotherapy and salicylic acid in different concentrations are relatively low (45.61% and 13.6%, respectively) when compared to alternative therapies such as laser, cantharidin-podophyllin-salicylic acid master formulation (CPS formulation) (97.81%), or topical antivirals (72.45%) [6]. Moreover, these treatments can injure the surrounding healthy skin, leading to pain and functional limitations in the patient [6,7,11]. On the other hand, more invasive treatments, such as the CPS formulation, immunotherapy, and intralesional bleomycin, are not indications for plantar warts by organisms such as the Food and Drug Administration (FDA) or the Spanish Agency of Medicines and Health Products (AEMPS) [6].

Nitric acid is a commonly used treatment for plantar warts employed by podiatrists. This potent caustic agent destroys tissue by denaturation [12]. Treatment is often conducted empirically with a 60% diluted nitric acid, as the optimal acid application amount is not yet standardized or protocolized [13]. NZCS, a novel medical device containing organic acids, nitric acid, and zinc and copper salts, can be applied to warts; this induces a painless caustic effect and causes mummification of the impacted tissue [14,15]. Cusini et al. [16] saw a 90% complete wart cure rate following four NZCS applications in a patient series suffering from common warts; only two of the 37 patients had plantar warts. Giacaman et al. [17] reported a total cure rate of 87.5% for periungual warts and 71% for palmoplantar warts after an average of six NZCS applications in a case series that included children aged 4–16: 24 periungual warts, two palmoplantar warts, and five plantar warts (16.12% of cases). A recent clinical trial compared the efficacy of NZCS and cryotherapy in treating plantar warts and found a cure rate of 56.6% (13 of 23 patients) with NZCS and 65.5% (19 of 29 patients) with cryotherapy [18].

Hence, the goal of this study is to analyze the healing rate of NZCS in a series of plantar wart cases. Additionally, our secondary objectives include determining the minimum number of product applications necessary for healing and the time frames of healing to protocolize and standardize its use.

## 2. Material and Methods

### 2.1. Participants and Simples

This study was conducted in compliance with the Declaration of Helsinki and current national laws governing research involving patients [19]. Informed consent was not necessary due to the retrospective nature of this study.

A descriptive study was conducted involving patients exhibiting symptoms of plantar warts who underwent chemical treatment with a nitric–zinc complex. To accomplish this, medical records were gathered from a specialty clinic focused on plantar wart management between January 2016 and December 2022.

The inclusion criteria for this study included both male and female patients over the age of 12, who had undergone treatment for one or more plantar warts with one or more applications of a zinc nitric complex. Patients without a confirmed diagnosis of HPV plantar wart via a polymerase chain reaction (PCR) test before treatment were not included. Wart samples were obtained using a scalpel to scrape the hyperkeratotic surface of the lesion and then collected in an Eppendorf tube, adhering to the same method as described in our 2022 publication [3]. The protocol we followed to identify HPV and its types was the same as in our previous studies on sampling methods and virus genotypes [3,4]. In addition, patients with immune system diseases such as HIV or autoimmune diseases were excluded from this study.

**Figure 1:**
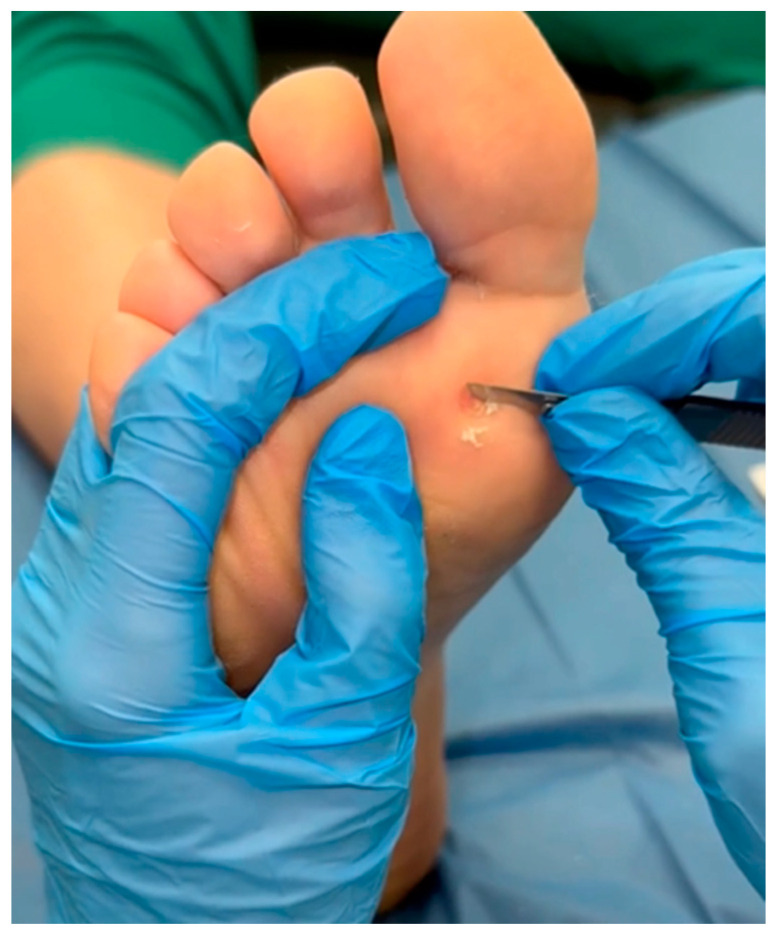
Debridement of the hyperkeratotic tissue covering the wart.

**Figure 2:**
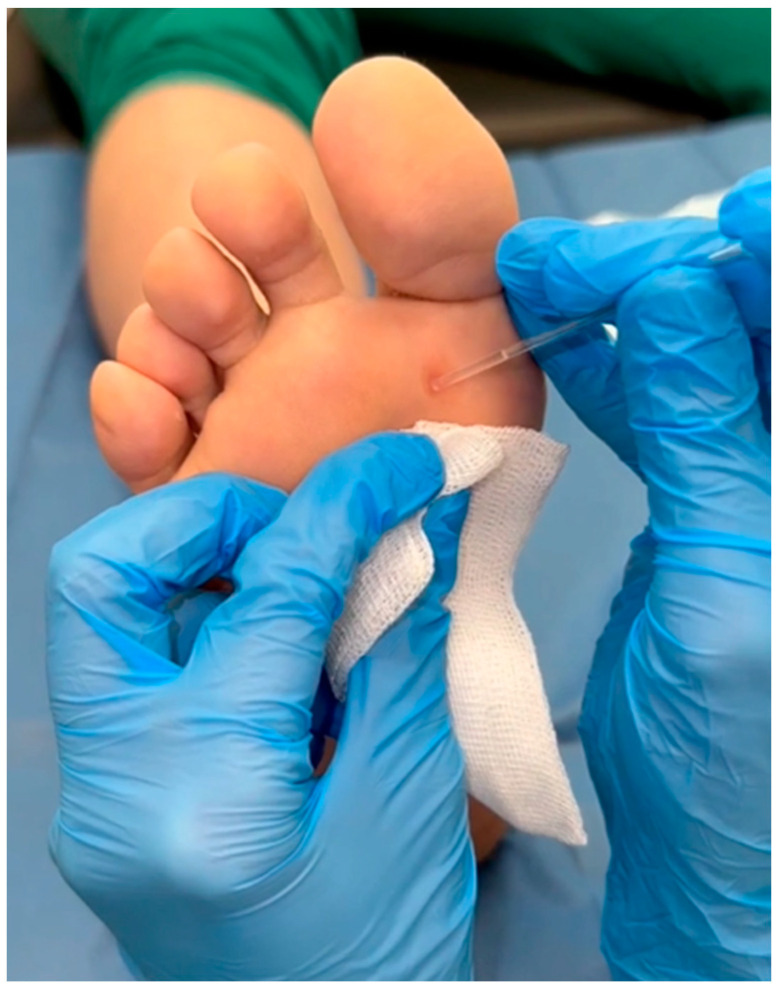
Application of the nitric–zinc complex on a plantar wart.

NZCS was topically applied with Verrutop^®^ (Isdin, Barcelona, Spain). Verrutop^®^ is an aqueous solution composed of organic acids (lactic, oxalic, and acetic acids), nitric acid, along with zinc and copper salts. It is indicated for the treatment of cutaneous warts. Before each treatment, the wart’s hyperkeratotic tissue was debrided using a No. 3 scalpel handle and a No. 15 scalpel (Figure 1) [20]. The product was then applied in this manner: the Verrutop^®^ ampoule (0.1 mL) was broken first, and the solution was loaded into one of the provided product applicators [18]. It was subsequently applied by directly contacting the entire lesion surface with small touches (Figure 2) [18]. Lastly, the treated area was covered using a non-adhering dressing. Patients were instructed to clean the area daily with 70° alcohol to reactivate the product until the next weekly check-up [18].

In addition to the primary variable, which was the healing of plantar warts (yes/no), the following secondary variables were collected: age, sex, duration of the condition (in months), number of plantar warts, location, HPV biotype, type of lesion (endophytic/exophytic), previous treatment (yes/no), type of previous treatment, number of NZCS applications, duration of NZCS application (in weeks), and time (in weeks) for complete healing and recurrence.

Clearance of the plantar wart was defined as the absence of clinical signs of the wart and the restoration of normal skin. When a patient showed signs of plantar wart after healing and clinical discharge, it was considered a recurrence of the wart.

### 2.2. Statistical Analyses

Statistical analysis was conducted using SPSS v.22 (IBM Corp. SPSS Inc., Chicago, IL, USA), with frequency and descriptive analyses performed.

The Kolmogorov–Smirnov test was used to determine whether the variables followed a normal distribution (*p* > 0.05), which would warrant the application of parametric tests, or if they displayed a non-normal distribution (*p* < 0.05), necessitating nonparametric tests. According to the Kolmogorov–Smirnov test results, all the quantitative variables analyzed exhibited a non-normal distribution (*p* < 0.05). Consequently, nonparametric tests were utilized for their analysis.

The logistic regression model was used to analyze qualitative and quantitative variables. For the comparison of other types of variables, the χ^2^ test was employed with qualitative variables, while the Mann–Whitney U test was used when dealing with one qualitative and one quantitative variable. A confidence level of 95% was designated, and statistical significance was set at *p* < 0.05.

The required minimum sample size for this analysis was estimated to be 39 samples, based on a statistical power of 0.80 and an alpha level of 0.05, using the GRANMO Sample Size Calculator version 7.12 Online (Institut Municipal d’Investigació Mèdica, Barcelona, Spain).

## 3. Results

Finally, 72 patients suffering from plantar warts were enrolled in this study. Table 1 presents the basic characteristics of the participants included in this study.

The length of time the warts had evolved at the time of the clinic visit was only available for 26 patients, with the most common location being the plantar area of the forefoot (63.9%) (Table 2). The type of wart (endophytic/exophytic) was only documented for 13 patients (Table 2).

The most prevalent biotype was HPV1 (40.3%), followed by biotype 5 (16.7%) and biotype 14 (9.7%). The prevalence of the remaining biotypes can be found in Table 2.

Sixteen patients had been previously treated by another medical professional (podiatrist and/or dermatologist) without the resolution of their warts. The type of pre-treatment in these 16 patients included nitric acid in 12 patients (75%), salicylic acid in two patients (12.5%), a CPS formulation (1% cantharidin, 5% podophyllin, and 30% salicylic acid) in one patient (6.25%), and cryotherapy with liquid nitrogen in one patient (6.25%).

**Figure 3:**
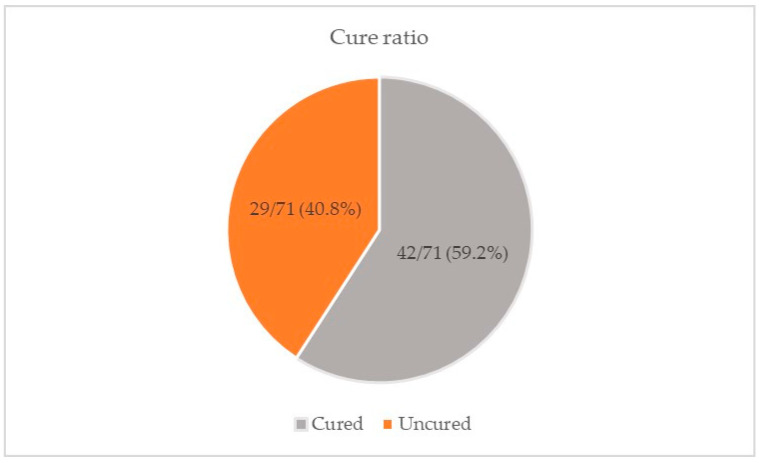
Cured patients with NZCS (%).

The cure rate was documented for 71 of the patients involved in this study. The cure rate associated with NZCS was 59.2% (Figure 3). The average number of NZCS applications was 5.9 ± 3.0, while the mean length of the treatment was 9.4 ± 7.1 weeks.

Table 3 displays the results of the univariate analysis. A statistical association was found between the healing of plantar warts and the subsequent variables: virus biotype, evolution time, and age.

Subsequently, we performed a multivariate model with the significant variables from the univariate analysis (*p* < 0.05). It showed no association between the variables and wart healing.

Additionally, a recurrence rate of 6.7% (4/72) was observed in patients treated with NZCS during the chart review.

## 4. Discussion

In this study, we observed a 59.2% cure rate for the NZCS as a treatment for plantar warts. Notably, there is only one clinical trial, published in 2023, that has evaluated the efficacy of the NZCS compared to first-line treatment with liquid nitrogen cryotherapy for plantar warts [18]. This trial concluded with only 23 patients completing the follow-up post-NZCS treatment. García Oreja et al. [18] reported a complete cure rate for plantar warts of 56.6% (13/23) with NZCS and 65.5% (19/29) with cryotherapy. Notably, they found no statistically significant differences between the two groups. The cure rates for NZCS observed in this study (59.2%) and in the randomized control trial by García Oreja et al. [18] are lower than those found in the case series published by Giacaman et al. [17] and Cusini et al. [16]. These authors reported NZCS cure rates for plantar warts ranging from 71% to 90% [16,17]. However, the sample of plantar warts included in these studies was only five and two warts, respectively [16,17].

The complete cure rates observed in this study with NZCS exceeded the cure rates reported in recent publications examining the efficacy of topical treatments for plantar warts. This trend was evident in the clinical trial by Cockayne et al. [21], which indicated a plantar wart cure rate of 14.3% for the group treated with liquid nitrogen cryotherapy and 13.6% for those treated with 50% salicylic acid. García Oreja et al.’s [6] systematic review, published in 2020, revealed that average cure rates for plantar warts were higher with cryotherapy (45.61%) than with salicylic acid (13.6%), but these underperformed relative to second-line treatments such as CPS formulation (97.82%), immunotherapy (68.14%), laser (79.36%), topical antivirals (72.45%), and intralesional bleomycin (83.37%). Another systematic review, published by Hekmatjah et al. [10], synthesized large interventional and observational studies, each involving more than 100 patients per study. This review concluded that despite their decreased efficacy, cryotherapy and salicylic acid persist as the first-choice treatments, even with the emergence of innovative treatments such as laser and intralesional immunotherapy.

In the logistic regression model analysis, there was no observed significant association between cure and any of the variables analyzed. Potential confounding variables such as HPV biotype, time of evolution, and age were taken into account due to their significant result in the univariate model (Table 3). Contrarily, García Oreja et al. [18] found an association between the average number of applications and cure, which was lower in cured patients (3.28 ± 1.63) than in uncured patients (5.5 ± 1.27). Moreover, other studies have identified an association between the virus biotype and the likelihood of cure or treatment resistance of warts. Bruggink et al. [22], in a randomized controlled trial, discovered that plantar warts with HPV 1 had a better natural history than those with other biotypes. They concluded that both salicylic acid and cryotherapy had better results than no treatment on warts with HPV1. However, with HPV2, 27, and 57 warts, only salicylic acid proved more effective than no treatment, while cryotherapy did not. In a separate multicenter randomized controlled trial, Hogendoorn et al. [2] found that plantar warts positive for HPV2 or HPV27 were more resistant to treatment than those containing HPV1. More recently, García Oreja et al. [4] reported a statistical association between biotype and the likelihood of cure, spontaneous resolution, and recurrence of treated plantar warts, with HPV 1 presenting the highest probability of any of these three incidents.

The findings of this study could have significant clinical relevance, not only due to the observed cure rates with NZCS but also because the product contains nitric acid, a substance previously used empirically by healthcare professionals to treat warts. Moreover, it is noteworthy that, to the best of our knowledge, this study has the largest sample size of patients with plantar warts treated with NZCS.

This study possesses several limitations. Being a retrospective study, not all variables could be collected for every patient due to their unavailability in the clinical history. Moreover, given the retrospective nature of this study, there existed no protocol concerning the number of applications and patient follow-up times. All patients received treatment in the same specialized clinic for plantar warts, which leaves uncertainties on whether the results might differ in other centers or different patient populations. This is not a clinical trial, so there was no comparison group or randomization, potentially leading to bias in the results. Furthermore, no documentation of possible adverse reactions or complications from the treatment, such as wounds or blisters, was recorded.

The authors of this study conclude that the NZCS is an effective treatment for plantar warts, which can be used as a first-line treatment in the general population. Therefore, future studies analyzing the efficacy and safety of its use in different populations may be useful in clinical practice.

## Figures and Tables

**Table 1:** Baseline characteristics of participants and their plantar warts.

Characteristics	
Nº (%) of each sex	
Female	50 (69.4)
Male	22 (30.6)
Age (years)	
Mean (SD)	49.2 (18.7)
Median (range)	53.0 (11.0–77.0)

**Table 2:** Characteristics of plantar warts.

Characteristics	Nº of Plantar Warts (%)
Duration of warts	
1–6 months	8 (11.1)
7–12 months	3 (4.2)
>12 months	15 (20.8)
Location	
Plantar forefoot	46 (63.9)
Plantar heel	9 (12.5)
Fingertips	12 (16.6)
Interdigital	1 (1.4)
Other locations	4 (5.6)
Type of wart	
Endophytic	10 (13.9)
Exophytic	3 (4.2)
HPV biotype	
1	29 (40.3)
5	12 (16.7)
2	7 (9.7)
14	7 (9.7)
65	4 (6.6)
57	3 (4.2)
24	3 (4.2)
19	2 (2.8)
27	2 (2.8)
111	2 (2.8)
Non-typable	1 (1.4)
Previous treatment	16 (22.2)

**Table 3:** Results univariate model.

Variables	Cured	Uncured	All Participants
	Frequency (%)	Frequency (%)	*p*-Value
Sex:			0.3
Female	27 (64.3)	22 (75.9)	
Male	15 (35.7)	7 (24.1)	
Wart location:			
Plantar forefoot	26 (61.9)	19 (65.5)	0.34
Plantar heel	6 (14.3)	3 (10.3)	
Fingertips	9 (21.4)	3 (10.3)	
Interdigital	0 (0.0)	1 (3.4)	
Other locations	1 (2.4)	3 (10.3)	
Type of wart			
Endophytic	5 (11.9)	5 (17.2)	0.07
Exophytic	0 (0.0)	3 (10.3)	
HPV biotype:			
1	24 (57.1)	5 (17.2)	**<0.01**
5	4 (9.5)	8 (27.6)	
2	4 (9.5)	2 (6.9)	
14	3 (7.1)	4 (13.8)	
65	4 (9.5)	0 (0.0)	
57	0 (0.0)	3 (10.3)	
24	0 (0.0)	3 (10.3)	
19	2 (4.8)	0 (0.0)	
27	0 (0.0)	2 (6.9)	
111	0 (0.0)	2 (6.9)	
Non-typable	1 (2.4)	0 (0.0)	
Time of evolution:			**0.04**
1–6 months	7 (16.7)	1 (3.4)	
7–12 months	0 (0.0)	3 (10.3)	
>12 months	10 (23.8)	4 (13.8)	
Previous treatment			
Yes	6 (16.2)	9 (36.0)	0.07
No	31 (83.8)	16 (64.0)	
Type of previous treatment:			0.18
Nitric acid	6 (16.7)	6 (22.2)	
Salicylic acid	0 (0.0)	2 (7.4)	
CPS formulation	0 (0.0)	1 (3.7)	
	Mean (SD)	Mean (SD)	*p*-Value
Age	45.3 (19.6)	55.5 (15.7)	**0.02**
Nº of applications	5.8 (3.0)	6.0 (3.0)	0.77

Note: Bold value statistically significant *p* < 0.05.

## Data Availability

The data that support the findings of this study are available from the corresponding author upon reasonable request.
